# Apoptosis of supraoptic AVP neurons is involved in the development of central diabetes insipidus after hypophysectomy in rats

**DOI:** 10.1186/1471-2202-9-54

**Published:** 2008-06-25

**Authors:** Yihua Wang, Cuiping Zhao, Zhigang Wang, Chengwei Wang, Wenfeng Feng, Lijin Huang, Jialin Zhang, Songtao Qi

**Affiliations:** 1 Department of Neurosurgery, Nanfang Hospital of Nanfang Medical University, Guangzhou 510515, Guangdong, PR China; 2 Department of Neurosurgery, Second Hospital of Shandong University, Jinan 250033, Shandong, PR China; 3 Department of Neurology, Second Hospital of Shandong University, Jinan 250033, Shandong, PR China

## Abstract

**Background:**

It has been reported that various types of axonal injury of hypothalamo-neurohypophyseal tract can result in degeneration of the magnocellular neurons (MCNs) in hypothalamus and development of central diabetes insipidus (CDI). However, the mechanism of the degeneration and death of MCNs after hypophysectomy in vivo is still unclear. This present study was aimed to disclose it and to figure out the dynamic change of central diabetes insipidus after hypophysectomy.

**Results:**

The analysis on the dynamic change of daily water consumption (DWC), daily urine volume(DUV), specific gravity of urine(USG) and plasma vasopressin concentration showed that the change pattern of them was triphasic and neuron counting showed that the degeneration of vasopressin neurons began at 10 d, aggravated at 20 d and then stabilized at 30 d after hypophysectomy. There was marked upregulation of cleaved Caspase-3 expression of vasopressin neurons in hypophysectomy rats. A "ladder" pattern of migration of DNA internucleosomal fragments was detected and apoptotic ultrastructure was found in these neurons. There was time correlation among the occurrence of diabetes insipidus, the changes of plasma vasopressin concentration and the degeneration of vasopressin neurons after hypophysectomy.

**Conclusion:**

This study firstly demonstrated that apoptosis was involved in degeneration of supraoptic vasopressin neurons after hypophysectomy in vivo and development of CDI. Our study on time course and correlations among water metabolism, degeneration and apoptosis of vasopressin neurons suggested that there should be an efficient therapeutic window in which irreversible CDI might be prevented by anti-apoptosis.

## Background

In the field of neurosurgery, disorders of the hypothalamic/posterior pituitary usually occur in humans after surgery of the hypothalamus and its proximal region. Abnormal water and electrolyte metabolism are typical postoperative complications. Injury to magnocellular vasopressin (AVP) and oxytocin (OT) neurons induces marked changes in the morphology and function of the neurohypophysis. Axotomy leads to neuronal retrograde degeneration in the peripheral and central nervous system [1-4]. In the hypothalamo-neurohypophyseal system (HNS), various types of axonal injury in vivo, including pituitary stalk compression[5,6], hypophysectomy[7], neurohypophy-sectomy [8-10] and pituitary stalk transection [11-13], result in degeneration of the magnocellular neurons of the hypothalamus and the development of diabetes insipidus. In addition, disruption of the axons of the HNS also leads to retrograde degeneration of substantial numbers of magnocellular neurons in the supraoptic (SON) and paraventricular (PVN) nuclei of the hypothalamus and leads to 74%–90% loss of the magnocellular neurons (MCNs) in the paraventricular (PVN). However, these studies only reported abnormal metabolism and degeneration of MCNs after axotomy but the time course and correlations between metabolism and histology of the degeneration of MCN are not clear which will facilitate us to find the therapeutic window.

Cell death is usually classified as apoptosis and necrosis which are differentiated on the basis of morphological abnormalities of cells at the ultrastructural level, patterns of DNA fragmentation on agarose gel electrophoresis. Apoptosis is characterized by membrane blabbing, perinuclear chromatin condensation, organelle swelling and by endonuclease-mediated internucleosomal DNA fragmentation into a "ladder" pattern. Necrosis is characterized by diffuse organelle swelling and lysis as well as random DNA fragmentation resulting in "smearing" of DNA on agarose gels [14-17]. Caspase-3 is considered the central apoptotic effector enzyme responsible for many of the biochemical and morphological features of apoptosis[18,19]. Activation of caspase-3 represents an irreversible step in the cell death pathway and cells containing activated caspase-3 are prone to die[20]. It has been reported that in organotypic cultures of the HNS, extensive cell death of MCNs die by apoptosis after the massive axotomy [21-23]. The neurotrophic factors, CNTF and LIF, and the neural activity can significantly reverse the cell death of the MCNs in vitro [21,23-26].

There are many studies to date have ascertained possible mechanism about the degeneration resulting from the axonal damage of CNS neurons. It has been reported that the cell death of MCNs in organotypic cultures in vitro is due to apoptosis. But the histopathological change and the type of death of MCNs are not clear after axotomy of HNS in vivo. This present study was aimed to investigate the time course and correlation between abnormal water and electrolyte metabolism and degeneration of MCNs as well as the mechanism of cells death of MCNs after hypophysectomy. Daily water consumption(DWC), daily urine volume(DUV), specific gravity of urine(USG) and plasma AVP concentration were measured; AVP-immunopositive neurons were counted at 3 d, 10 d, 20 d and 30 d after hypophysectomy and apoptosis were analyzed.

## Results

### Clinical findings

All rats in hypophysectomy group started drinking water after recovery of anesthesia and started eating food from the next day. No marked neurological abnormalities or respiratory symptoms appeared in any of the animals throughout the follow-up period and their activity and appetite were constant and normal.

### Changes of DWC, DUV and USG

**Figure 1:**
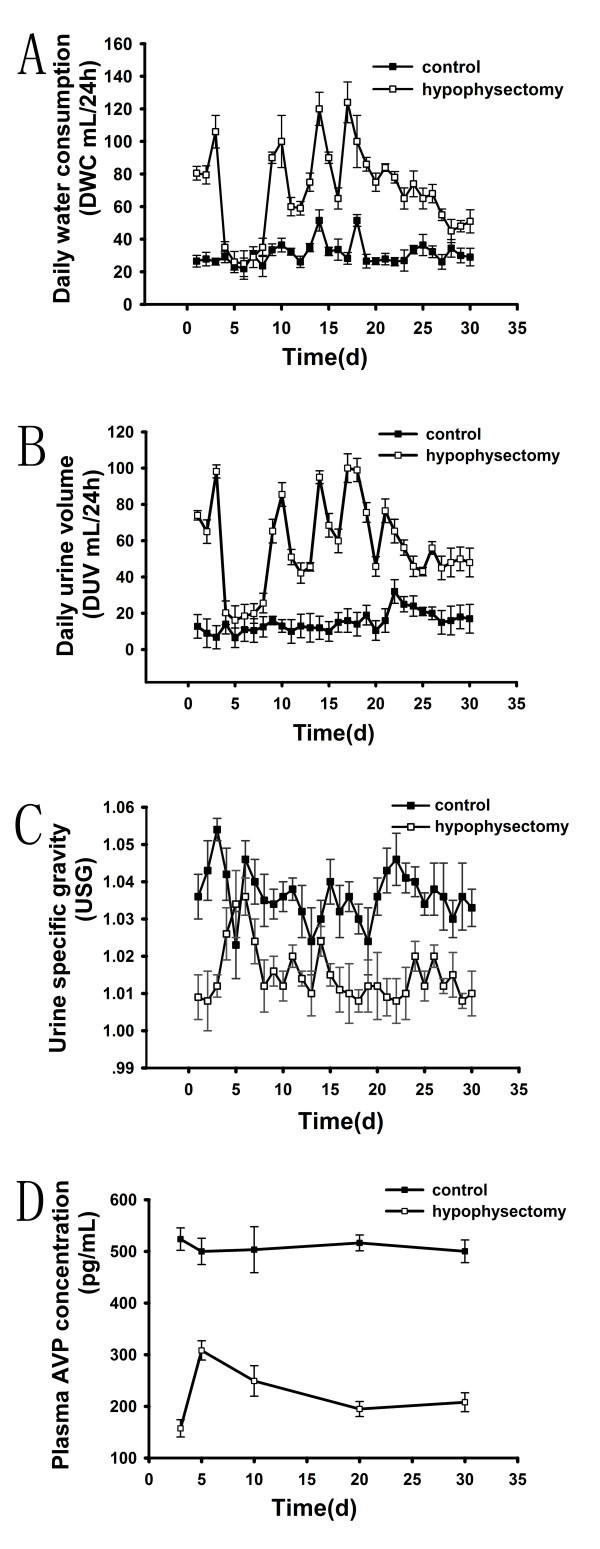
**Dynamic change of daily water consumption (DWC), daily urine volume (DUV), urine specific gravity (USG) and plasma AVP concentration after hypophysectomy**. DWC, DUV and USG remained relatively constant over the entire period of observation in control group while there was a triphasic pattern in hypophysectomy rats. Phase I: a sharply changes during the first 3 days after surgery; Phase II: a comparable low level with those in control group at 4–8 d; Phase III: increased of DWC and DUV and decreased USG again in the following days. Plasma AVP concentration in hypophysectomy group was less than that in control group statistically significantly.

The average daily water consumption (DWC), daily urine volume (DUV) and specific gravity of urine (USG) of control group were 31.1 ± 8.1 ml/24 h, 15.1 ± 5.9 ml/24 h and 1.036 ± 0.007 respectively, which remained relatively constant throughout the entire period of observation. Hypophysectomy rats exhibited a triphasic pattern of DWC, DUV and USG: a sharply increased DWC (88.7 ± 14.1 ml/24 h) and DUV (79.2 ± 15.7 ml/24 h) during the first 3 days after surgery (phase l), followed by low level DWC (30.1 ± 5.2 ml/24 h) and DUV (20.1 ± 3.8 ml/24 h) at 4–8 d which were comparable with those observed in control group (phase 2). After phase 2, DWC (76.4 ± 18.6 ml/24 h) and DUV (62.1 ± 14.8 ml/24 h) increased again and remained at an elevated level in the following days (phase 3)(Fig. 1A,B). The changes of USG also exhibited a triphasic pattern (1.010 ± 0.003 during the first 3 days, 1.026 ± 0.009 at 4–8 d and 1.013 ± 0.007 during the next time). (Fig. 1C)

### Change of plasma AVP concentration

Plasma AVP concentrations were relatively constant at various times in control group (Fig. 1D). Plasma AVP concentration was 157.4 ± 16.7 pg/ml at 3 d after surgery in hypophysectomy rats. The difference between two groups was statistically significant (*P *< 0.01). At 5 d after surgery, it was 308.2 ± 18.6 pg/ml in hypophysectomy group, which increased significantly compared with those at 3 d. In the follow-up period, plasma AVP concentration decreased again. (Fig. 1D)

### AVP-positive cell counts

**Figure 2:**
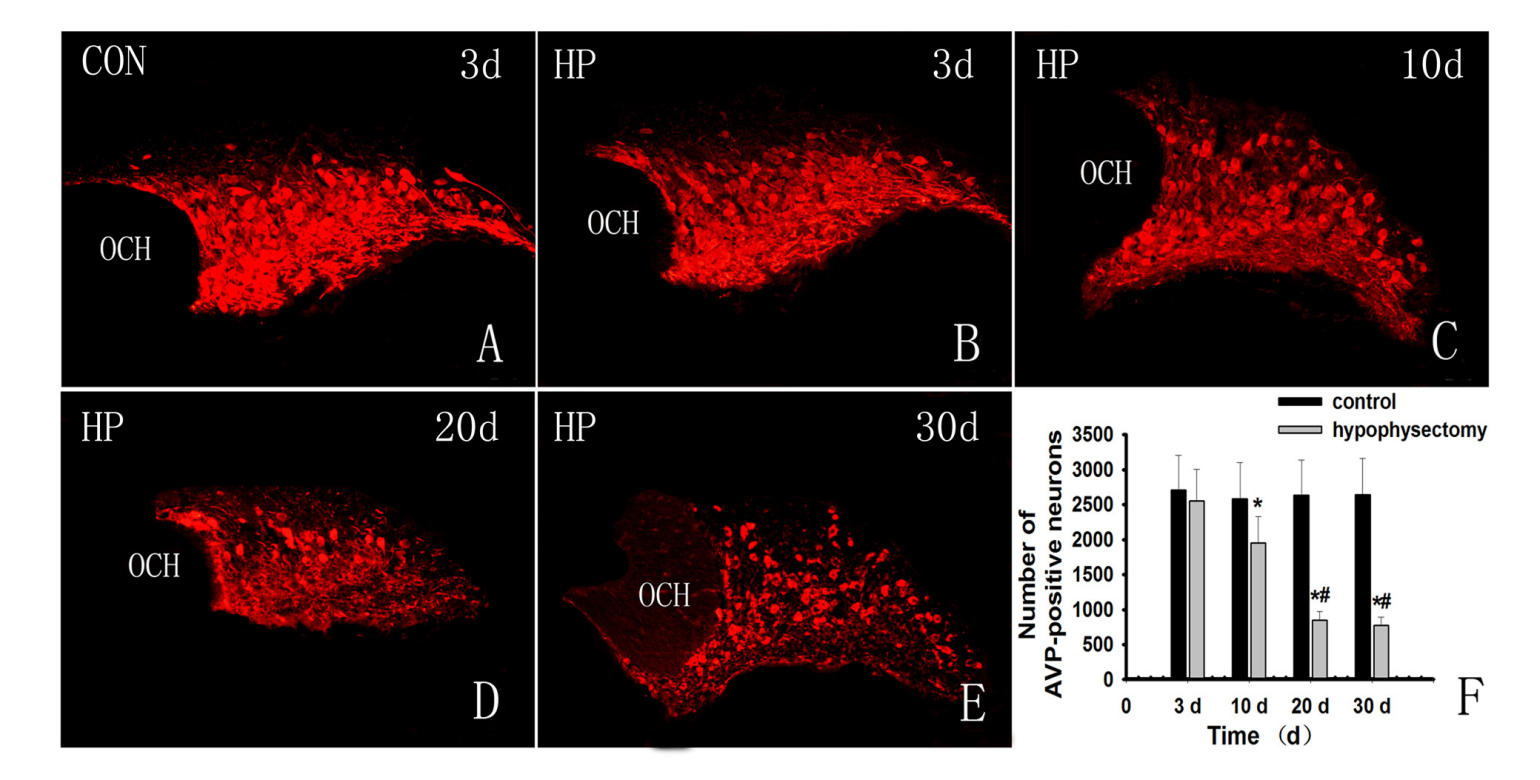
**Immunofluresence analysis of hapothalamic supraoptic AVP neurons**. A: There was no change in number of AVP-positive neurons in control group. B: AVP-immnopositive neurons at the 3 d after hypophysectomy. There was a bit decrease of AVP-positive cells. C, D and E: AVP-positive neurons at the 10 d, 20 d and 30 d respectively. The number of AVP-positive neurons decreased from the 10 d and reached minimum at 20 d and 30 d. F: AVP-positive cell counts. There were 93.2% of AVP-positive neurons survived at the 3 d after hypophysectomy,75.4% at the 10 d, 32.2% at 20 d and 29.2% at 30 d (*, P < 0.01 compared with the control group). The numbers of AVP-immunopositive neurons at 20 d and 30 d were significantly less than that at 10 d but there was no difference between those at the 20 d and 30 d (#, P < 0.01 compared with the 10 d).

Immunofluorescence using anti-AVP antibody was performed in the hypothalamic region and AVP-positive cells were counted in the supraoptic nuclei. In hypophysectomy group, the AVP-positive cell numbers in supraoptic nuclei of both sides were 2554.2 ± 379.6, 1949.2 ± 136.7, 847.2 ± 255.7 and 771.4 ± 202.6 cells at 3 d, 10 d, 20 d and 30 d respectively after surgery (Fig. 2B,C,D,E). Compared with the control group, there were 93.2%, 75.4%, 32.2% and 29.2% of survival AVP neurons in these nuclei at each time point respectively in hypophysectomy group. The number of AVP-positive neurons decreased progressively from the 10 d, and reached minimum at 20 d and 30 d (there was no significant difference between 20 d and 30 d). (Fig. 2F)

### Immunostaining of the active form of Caspase-3 and colocalized with AVP

**Figure 3:**
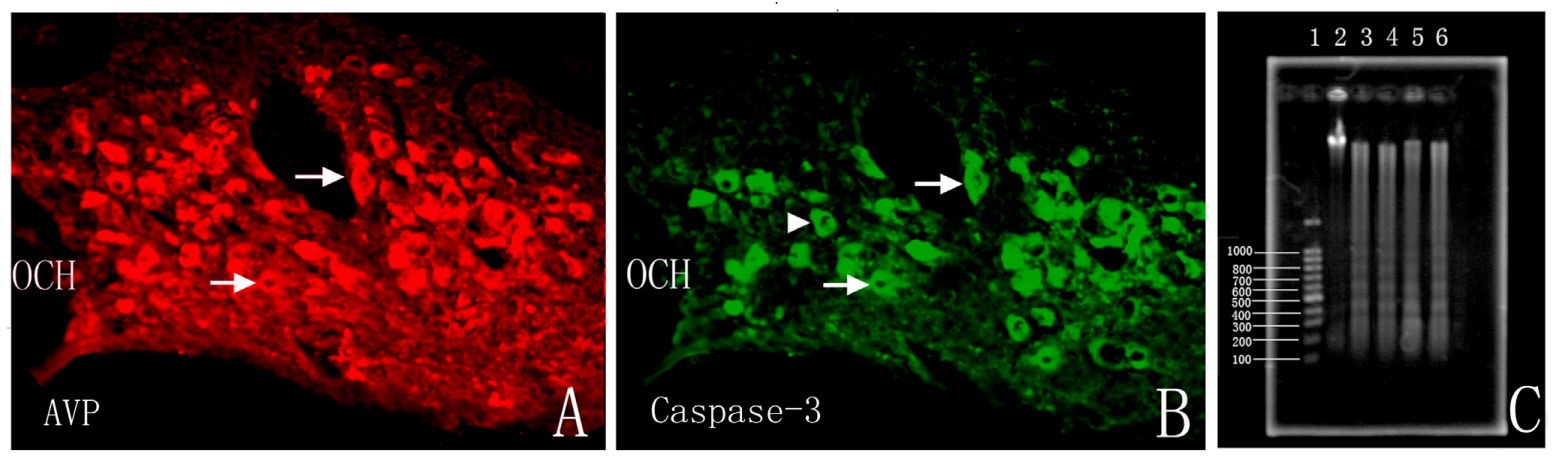
**Immunofluresence analysis of the active form of Caspase-3 and Patterns of DNA fragmentation.** A and B showed the AVP and Caspase-3 immunostaining of supraoptic nucleus in the same slide at 10 d after hypophysectomy. Generally, there were 793.6 ± 164.5 Caspase-3 positive cells (about 30.1% against the control group) at 10 d after hypophysectomy. The immunostaining of AVP in these same slides with Caspase-3 showed that most of Caspase-3 immunopositive cells were clearly visible colocalized with AVP-immunopositive neurons (arrowheads) and a few Caspase-3 immunopositive cells were not colocalized with AVP (arrow), which may be the OT neurons. C: Agarose gel electrophoresis of DNA samples purified from hypophysectomy group at 10 day after surgery showed oligonucleosomal bands in a "ladder" pattern of migration (lanes 3–6), whereas samples from the control animals did not yield it (lane 2) and line 1 showed the DNA markers.

Cleaved caspase-3 is the activated form of caspase-3, a critical effecter of apoptosis [27]. Immunofluorescence analysis using the specific antibody against cleaved caspase-3 was performed at 10 d after surgery. There were very few caspase-3 immunopositive neurons in control group. Marked increased caspase-3 expression was present in supraoptic nuclei of hypophysectomy rats. There were 793.6 ± 164.5 Caspase-3 immunopositive cells (about 30.1% against the control group) at 10 d after hypophysectomy. The double immunostaining of AVP/caspase-3 showed that most of Caspase-3 immunopositive cells were clearly visible colocalized with AVP-immunopositive neurons (arrowheads) and a few Caspase-3 immunopositive cells was not colocalized with AVP-immunopositive neurons (arrow), which might be OT neurons. (Fig. 3A, B)

### Patterns of DNA fragmentation

DNA from the tissues of hypothalamic supraoptic nucleus was isolated and agarose gel electrophoresis was performed. This method allowed the distinction between apoptotic "laddering" of DNA into internucleosomal fragments and necrotic "smearing" of DNA into random-size fragments. Agarose gel electrophoresis of DNA purified from hypophysectomy group at 10 d after surgery showed oligonucleosomal bands in a "ladder" pattern of migration, whereas samples from the control animals did not yield it (Fig. 3C).

### Ultrastructural Observations

**Figure 4:**
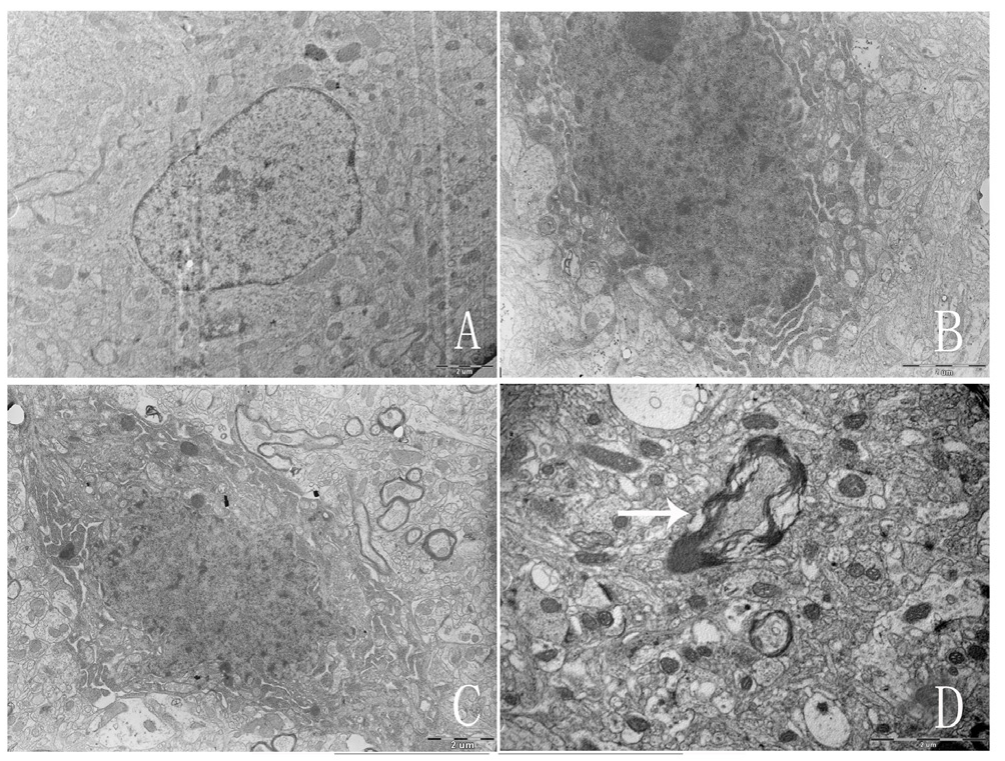
**Ultrastructure under electron microscopy.** Ultrastructural analysis revealed numerous neurons undergoing apoptotic degeneration in hypophysectomy group. A showed the normal cellular ultra-structures in control group. B-D showed apoptotic ultrastructures (B, C showed nuclear condensation and fragmentation. D showed degeneration of myelin.)

Ultrastructural analysis of the hypophysectomy group revealed numerous neurons undergoing apoptotic degeneration. The neurons of hypothalamic supraoptic nucleus showed several apoptotic features such as nuclear condensation and fragmentation, cell surface protrusions (Fig. 4).

## Discussion

In mammals, the majority of vasopressin producing magnocellular neurons and oxytocin cells distribute in the hypothalamic paraventricular and supraoptic nuclei [40]. These cells send long axonal projections toward the fenestrated capillaries of the neurohypophysis where they release their hormonal content into the blood stream to reach peripheral target organs. Circulating vasopressin, the antidiuretic hormone, maintains extracellular fluid balance by regulating water resorptions from the distal tubulus of the kidney and is one of the most potent vasoconstrictors yet identified [22]. Deficient secretion of vasopressin results in the syndrome of diabetes insipidus which is characterized by polydypsia and polyuria. Diabetes insipidus was one of the major complications of hypothalamo-neurohypophyseal stract damage and cerebral neurosurgery which manifested as abnormalities of electrolyte metabolism, such as central hypernatremia or central hyponatremia [28,29]. Hypophysectomy is a conventional animal model in the study of dibetes insipidus. Following neurohypophysectomy, rats exhibit a pronounced diabetes insipidus and dramatically decreased urinary levels of AVP [30]. Dohancics et al. reported that stalk-compressed rats exhibited a triphasic pattern of water intake[31]. As these previous reports, the present study exhibited a triphasic pattern of Daily water consumption(DWC), daily urine volume(DUV) and specific gravity of urine(USG) of hypophysectomy rats: an increased water intake and urine volume during the first 3 days after surgery (phase l), followed by a comparable of DWC and DUV with those observed in sham-operated rats 4–8 d (phase 2), after which DWC and DUV increased again and remained at an elevated level throughout the rest of time (phase 3). The changes of USG also exhibited a triphasic pattern. Diabetes insipidus is a kind of pathologic state resulting from decreased ability of the kidney to concentrate urine because of the decreased secretion of AVP from the posterior pituitary gland or resistance to the action of AVP. AVP is mainly secreted by large neurosecretory cells in the hypothalamic supraoptic and paraventricular nuclei. It descends along the nerve axon and is stored in the posterior pituitary gland through the inner layer of the median eminence. Total resection of the pituitary gland results in loss of storage and secretion capability for AVP in the posterior pituitary in animal. To confirm the correlation between the diabetes insipidus and plasma AVP concentration, plasma AVP concentration also was measured at3 d, 5 d, 10 d, 20 dand 30 d after surgery. In the present study, plasma AVP concentration was significant lower in hypophysectomy (157.4 ± 16.7 pg/ml) than that in the control group (523.7 ± 21.7 pg/ml) at the 5 d. At the 5 d after surgery, plasma AVP concentration was higher than that at the 3 d, but was still lower than that in the control. In the following time, plasma AVP concentration decreased again at 10 d, 20 d and 30 d after hypophysectomy. Plasma AVP concentration also exhibited a triphasic pattern in accord with the pattern of diabetes insipidus. Many previous studies reported that the plasma AVP concentration decreased after axotomy and an ectopic neurosecretory gland-like structure above the pituitary was found as well as an ectopic miniature neurohypophysis at the distal end of the pituitary stalk formed after hypophysectomy, neurohypophysectomy, stalk section or mechanical stalk compression. In this present study plasma AVP concentration decreased at 3 d after hypophysetomy was due to loss of storage and secretion capability for AVP in the posterior pituitary. The plasma AVP concentration relatively increased at 5 d probably because of the release of the AVP stored in the magnocellular neurons through the ectopic miniature neurohypophysis while the decreased concentration of AVP in the following time points resulted from the degeneration of magnocellular neurons of hypothalamus.

Retrograde degeneration of magnocellular neurons is well known to occur after hypophysectomy[7], neurolobectomy [8-10] and stalk damage[13]. Although magnocellular degeneration after axonal damage has also been demonstrated quantitatively[8,31], these studies did not evaluate the time correlation between the degeneration of AVP neurons and the occurrence of diabetes insipidus. Immunofluorescence analysis revealed that AVP-positive cells tended to decrease after hypophysectomy, suggesting that excision of the posterior lobe resulted in the decrease of magnocellular neurons in the hypothalamus. Dohanics et al., used a different stereotaxic method to compression the stalk, which resulted in marked diabetes insipidus accompanied by the significant degeneration of the AVP neuron population in both SON and the PVN, resulting in the survival of only ~35% and ~27% of AVP neurons in these nuclei at 21 days after surgery, respectively[31]. This finding was similar to Herman's study [9,30], in which a marked loss of magnocellular AVP-containing neurons was induced after neurohypophysectomy in conjunction with chronic vasopressin agonist treatment. The present study demonstrated the survivals of 32.2% and 29.2% AVP neurons at the 20 d and 30 d after hypophysectomy, respectively (P > 0.05 between these two time-points), and the survivals of AVP neurons were 97.2% at the 3 d (P > 0.05 compared to the control group) and 75.4% at the 10 d (P < 0.01). These findings showed that the degeneration occurred from the 10 d after hypophysetomy, aggravated at the 20 d and then stabilized. This study showed that the AVP neurons did not begin to degenerate at the 3 d but diabetes insipidus occurred and plasma AVP concentration decreased, which was probably because the loss of AVP storage and secretion capability by resection of the posterior pituitary and an ectopic neural lobe had not been formed. An ectopic neural lobe formed after axotomy would enhance secretion of AVP into the portal circulation. At the 5 d after surgery AVP stored in the magnocellular neurons in the hypothalamus nuclei was released by the ectopic neural lobe and the plasma AVP concentration increased. From 10 d plasma AVP concentration decreased and diabetes insipidus occurred again because of the significant degeneration of AVP neurons. In one word, there was tight time correlation between the occurrence of diabetes insipidus, plasma AVP concentration and the degeneration of AVP neurons after hypophysectomy.

Cell death is usually classified as apoptotic or necrotic based on biochemical and morphologic criteria [32-34]. Apoptosis and necrosis can be distinguished histologically[14]. Tissue necrosis is typified by loss of membrane integrity, morphological signs of organelle damage, nuclear flocculation, loss of lysosomal contents, cellular swelling and uncontrolled cell lysis [35,36]. Apoptosis is characterized by preservation of membrane integrity, cytoplasmic and nuclear condensation, diminution of cellular volume, plasma membrane bleb formation and morphological preservation of organellar structure. The cell eventually fragments into apoptotic bodies that are engulfed by neighboring cells and degraded [35,37,38]. During apoptosis, morphological changes often are accompanied by internucleosomal cleavage of genomic DNA [39,40]. In contrast to necrosis, apoptosis does not result in loss of cellular content and does not initiate an inflammatory response. Many lines of evidence in the previous studies indicate that the central neurons commit themselves to apoptotic death after axotomy. At the ultrastructural level the morphological features of these cells are associated classically with apoptotic death [14]. The selective degeneration of vasopressin neurosecretory neurons has been reported in cases of human diabetes insipidus[41,42] and in animal models after axotomy[30,31]. In the vitro studies, selective death of these neurons were also demonstrated in organotypic cultures of the hypothalmic nucleus and the nature of the death is apoptosis which could be specifically prevented by the administration of the cytokines CNTF[21,23-26]. It was found that the massive degeneration of vasopressin-positive neurons by apoptotic cell death was specific for the vasopressin magnocellular population. This present study was aimed to determine whether the degeneration of vasopressin neurons in supraoptic nuclei after hypophysectomy was due to apoptosis in vivo. The ultrastrctural changes of magnocellular neurons were observed at 10 d after hypophysectomy under electron microscope. Cytoplasmic condensation and blebbing, compaction and fragmentation of nuclear chromatin and the eventual dissolution of membranous barriers between organelles and cytoplasm were found. Fragmentation patterns of DNA isolated from hypothalamic supraoptic nuclei tissues using agarose gel electrophoresis was analyzed. This method allows the distinction between apoptotic "laddering" of DNA into internucleosomal fragments and necrotic "smearing" of DNA into random-size fragments[43]. DNA laddering is a common early step to all types of cell death. The initial event in cell death appears to be the activation of endonuclease(s) that generates a ladder of DNA fragments of high molecular weight. In apoptosis, this degradation goes uninterrupted until most of the DNA is fragmented into mononucleosomes, which are DNA fragments protected from further endonuclease digestion by intact histone octamers. In contrast, release of proteases from disrupted lysosomes follows at some time after endonuclease activation in necrosis leading to degradation of the protective histones and full exposure of the DNA to the endonuclease. This exposure results in random DNA degradation seen as a "smear" on agarose gel electrophoresis. In the present study, the typical DNA laddering was detected with agarose gel electrophoresis. It has been showed that the activation of caspase-3, followed by cleavage of specific substrates, may contribute to the process of apoptosis by structural changes or by affecting certain signaling molecules. Cleaved Caspase-3 is an important regulator of apoptosis and its proteolytically cleaved form is known to be upregulated in neurons undergoing apoptosis [44,45]. In our study, immunofluorescence analysis using the specific antibody against cleaved caspase-3 revealed that there was very low level of caspase-3 immunoreactivity in supraoptic neurons in control while caspase-3 was found progressively upregulated in hypophysectomy rats. To confirm the cell type of apoptotic neurons, vasopressin/caspase-3 double staining was performed. It was found that most caspase-3 immunopositive cells were colocalized with vasopressin. This present study suggested that the degeneration of vasopressin neurons was due to apoptosis in hypophysectomy rats.

## Conclusion

This study firstly demonstrated that apoptosis was involved in degeneration of vasopressin neurons in hypothalamic supraoptic nuclei after hypophysectomy in vivo and development of centrarl diabetes insipidus. These data suggested that anti-apoptosis would ameliorate the degeneration and protect these neurons after hypophysectomy. It has been reported that CNTF, Bcl-xL and Caspase inhibitor could increase the survival of rat vasopressin magnocellular neurons in organotypic cluture. It will be worth further exploring whether such trophic factors and apoptosis inhibitor would protect these neurons in vivo. Moreover our study on time course and correlations among metabolism, degeneration and apoptosis of MCN suggested an efficient therapeutic window in which irreversible diabetes insipidus might be prevented by antiapoptosis.

## Methods

### Animals

Adult malt Sprague-Dawley rats weighing 250–275 gm (Nanfang medical university, Guangzhou, China) were housed individually in wire-mesh cages in a temperature-controlled room (21–23°C) with lights on from 7:00 A.M. to 7:00 P.M. Animals were fed solid food or liquid diet as described below. All animal experiments were performed according to institutional guideline that is in compliance with national and international law and policies.

### Hypophysectomy and treatment

Hypophysectomy was performed by removing both the anterior and posterior pituitary by the parapharyngeal approach [46,47]. Briefly, animals were anesthetized with intraperitoneal injections of ketamine (80 mg/kg) and xylazine (8 mg/kg) and mounted upside-down in a stereotaxic apparatus. The skin on the ventral aspect of the neck was incised and the infrahyoid musculature separated on the midline and retracted to either side. The sella turcica was approached by blunt separation and a hole was drilled at the basal occipital suture to allow visualization of the pituitary gland. Anterior and posterior pituitary were removed by suction. The aspirated anterior and posterior pituitaries were examined to confirm the completeness of hypophysectomy. The wound was packed with gelfoam and the skin incision closed with suture. The animals in the control group received sham operation, which involved the entire surgical procedure with the exception of hypophysectomy. These rats had access to either solid chow ad libitum or 40 ml of liquid diet daily. They also had unrestricted access to tap water. After operation, daily water consumption (DWC), daily urine volume (DUV) and specific gravity of urine (USG) were measured in both groups. Blood was collected at 3 d, 5 d, 10 d, 20 d and 30 d after surgery(n = 20). The plasma AVP concentration was measured by the RIA method (Department of physiology, second military medical university, Shanghai, China) at each time point(n = 4).

### Tissue immunofluorescence analysis

At 3 d, 10 d, 20 d and 30 d after surgery, rats (n = 4) received a lethal dose of ketamine and xylazine. After an intracardiac injection of heparin (300 U), rats were perfused through the ascending aorta with 0.9% NaCl containing 2% sodium nitrite (total volume 200 ml per animal). The perfusion solution was then switched to a fixative consisting of 4% paraformaldehyde and 1.4% picric acid in 0.1 M phosphate buffer (PB, total volume 100 ml per animal). Then the rats were decapitated and the brains and pituitaries were stored in 25% sucrose at 4°C until sectioning. Hypothalami were cut with a freezing microtome into 25 μm sections in the coronal plane. Frozen sections of hypothalamus were collected in wells containing phosphate-buffered saline (PBS). After rinsing, alternate sections were treated for fluorescence immunostaining. The slides were washed with deionized water to remove the freezing medium, rinsed 3 times in PBS and permeabilized with 0.3% Triton X-100 in PBS at room temperature. Nonspecific antibody binding was blocked with incubation in 10% normal goat (Boster Co, WuHan, China) for 1 hour at room temperature. The slides were incubated overnight at 4°C with rabbit IgG polyclonal antibodies against arginine vasopression AVP (1:100, Phenix pharmaceuticals, CA, USA) followed by 2 hours of incubation with secondary antibody conjugated to Cy3 (1:100, Santa Cruz, CA, USA). The slides that were collected from 10 d after surgery continued to rinse and incubate overnight at 4°C in the second primary antibody monoclonal anti-Caspase 3 (1:200, Santa Cruze, CA, USA). Then the slides incubated in secondary antibody conjugated to FITC for 1 hour at room temperature. After rinsing them in PBS, the slides were counter stained with AVP and examined under fluorescent microscope (Olympus BX51, Olympus Co., Tokyo, Japan). Pituitaries were stored in 25% sucrose for 24 hours at 4°C and then immersed in 15% gelatin for 15 min at 37°C. Gelatin-embedded pituitaries were fixed in the perfusion solution for 24 hours at 4°C and then stored in 25% sucrose for 24 hours at 4°C. Pituitaries were cut with a freezing microtome into 40 μm sections. Sections were stained for AVP immunoreactivities. Rats with incomplete hypophysectomy (as determined by pituitary histology) were excluded from further evaluation.

### Cell counting

AVP and Caspase-3 immunopositive cells in the SON were counted on all sections. Neurons were counted at 40× magnification by identifying them visually or by visual identification combined with mapping of the position of identified neurons. The position of individual neurons was recorded using a microscope stage connected to a computer that recorded the x-y coordinates of the stage position. In each group, neurons in sections were counted over an 80 × 80 μm^2 ^area of the SON (n = 4). Raw counts were corrected for double-counting errors using Abercrombie's method.

### Ultrastructure under electron microscopy

The animals (n = 4) were anesthetized with ketamine and xylazine (i.p.) at 10 d after surgery and perfused with 150 ml of fresh 1% paraformaldehyde including 0.1% glutardldehyde in 0.1 M phosphate buffer (pH 7.4), followed by 350 ml of 2% paraformaldehyde with 2% glutaraldehyde in same buffer. The brain was postfixed overnight at 4°C in the latter fixative. The hypothalamic supraoptic nucleus tissue was subdissected into 1 mm^3 ^blocks washed in phosphate buffer and then treated with 2% osmium tetroxide in 0.1 M phosphate buffer for 2 hours at RT. Blocks were washed in the same buffer, dehydrated and embedded in Epon. Thin sections (gold interference color) were cut, stained with uranyl acetate and lead citrate and viewed with a Hitachi H7500 electron microscope (Hitachi, Tokyo, Japan).

### Analysis of DNA fragmentation

To detect oligonucleosomal DNA fragmentation as a marker of apoptosis, DNA was isolated from hypothalamic supraoptic nucleus tissues of control rats and hypophysectomy animals at 10 d post surgery (n = 4). As previously reported in detail (Portera-Cailliau et al.,1995), Genomic DNA was isolated by digesting the supraoptic nucleus tissue in digestion buffer (0.25 mg/ml proteinase K in 10 mM Tris, pH 7.5y10 mM EDTAy0.5% N-lauroylsarcosine) followed by extraction with phenolychloroform. Samples (20 mg) of DNA were resolved by 1% agarose gel electrophoresis.

### Data analysis

All results are expressed as mean ± SE. Statistical analysis was performed on logarithmic transforms of the data using ANOVA followed by Tukey's protected t tests for multiple comparisons where appropriate.

## Abbreviations

AVP: vasopressin; OT: oxytocin; SON: supraoptic nucleus; PVN: paraventricular nucleus; HNS: hypothalamo-neurohypophyseal system; CNS: central nervous system; MCNs: magnocellular neurons; PCD: programmed cell death; DWC: daily water consumption; DUV: daily urine volume; USG: specific gravity of urine; (USG)AC, PBS: phosphate buffered saline; CNTF: cliliary neurotrophic factor.

## Authors' contributions

YW and SQ designed the research. YW and JZ carried out the animal procedures and clinical observations. YW and CZ carried out the fluorescence analysis. YW, ZW and CW carried out the measurement of plasma vasopressin concentration. JZ, WF and LH participated in the ultrastructure and agarose gel electrophoresis analysis. YW, CZ and SQ participated in coordination and drafted the manuscript. All authors read and approved the final manuscript.
